# Situational simulation teaching can effectively enhance the clinical thinking ability of residents

**DOI:** 10.1186/s12909-025-08248-7

**Published:** 2025-11-25

**Authors:** Jing Meng, Li Wang, Danni Jin, Zihan Li, Qingqing Tao, Lan Ying

**Affiliations:** 1 Department of Critical Care Medicine, The Second Affiliated Hospital, Zhejiang University School of Medicine, Hangzhou, Zhejiang 310009 China; 2 Department of Emergency Medicine, The Second Affiliated Hospital of Zhejiang, University School of Medicine, No. 88 Jiefang Road, Shangcheng District, Hangzhou, Zhejiang Province 310052 China; 3 Department of Emergency, Shaoxing Second People’s Hospital, Shaoxing, 312000 China; 4 Department of Neurology, The Second Affiliated Hospital of Zhejiang University School of Medicine, Hangzhou, 31009 China

**Keywords:** Situational simulation teaching, Clinical thinking, Entrust able professional activities mini-clinical evaluation exercise

## Abstract

**Objective:**

To investigate the efficacy of situational simulation teaching (SST) in enhancing clinical thinking competencies among residents.

**Methods:**

A randomized controlled trial was conducted with second-year residents (*n* = 121) during their rotation (May-August 2025). Participants were allocated to either an intervention group (*n* = 57, receiving SST) or a control group (*n* = 64, conventional training). Pre- and post-intervention assessments evaluated clinical thinking performance. Faculty assessments using the entrust able professional activities (EPAs) and Mini-clinical evaluation exercise (Mini-CEX) were completed within 2 weeks post-training, supplemented by resident satisfaction surveys.

**Results:**

Both groups demonstrated significant post-intervention improvement in overall clinical thinking scores (all *P* < 0.05), except for the “Ethics and Professionalism” domain in controls (*P* = 0.12).The intervention group outperformed the control group in total clinical thinking scores and all sub-dimensions (*P* < 0.05), with higher EPAs scores, EPA pass rates, and Mini-CEX total scores (all *P* < 0.05). Resident satisfaction (content, resources, interaction, and teaching methods) was also significantly higher in the intervention group (*P* < 0.05).

**Conclusion:**

SST significantly enhances clinical thinking development in residency programs.

## Introduction

With the continuous advancement of medical education, clinical training methods for resident physicians are undergoing constant innovation. Traditional medical education primarily relies on classroom teaching and clinical practice, yet this “spoon-feeding” instructional model often fails to comprehensively enhance residents’ clinical thinking and emergency response capabilities. In this context, Situational simulation teaching (SST) has emerged as a transformative educational approach for resident development [1]. By replicating authentic clinical scenarios, this method effectively cultivates decision-making skills, teamwork competencies, and clinical techniques in complex environments, making it particularly valuable in high-stakes medical specialties [2, 3].Clinical thinking encompasses not only rapid patient assessment and diagnosis but also individualized treatment planning and multidisciplinary coordination. Enhancing these competencies is crucial for improving healthcare quality, reducing medical errors, and increasing patient survival rates [4].While simulation training has been adopted in medical education, its specific impact on the development of clinical thinking skills requires further investigation. This study examines the effects of integrating SST into resident education programs to address this evidence gap. The broader literature supports the role of simulation in improving clinical competencies. For instance, Ayed et al. demonstrated that emotional intelligence and critical thinking skills interact in nursing students, which suggests that simulation-based learning can simultaneously develop both cognitive and emotional competencies [5].

This study specifically aims to fill this gap by examining the effects of integrating SST into resident education programs, with a focus on emergency medicine in China. By utilizing validated assessment tools such as the Mini-CEX and Entrustable Professional Activities (EPAs), this research will provide more specific insights into how SST impacts the development of clinical thinking skills, and whether it leads to measurable improvements in residents’ decision-making, patient management, and team coordination under pressure. This focus on structured assessment will allow for a more rigorous evaluation of SST’s effectiveness in enhancing clinical thinking compared to previous studies that have often relied on more qualitative measures.

## Method

### Participants

This study was conducted at a single-center institution, with second-year residents rotating in the Department of Emergency Medicine from May to August 2025. Residents were randomly assigned to either the intervention group (*n* = 57) or the control group (*n* = 64) using a digital random number table. To ensure unbiased allocation, allocation concealment was implemented, meaning that the researchers responsible for enrollment and assignment were unaware of the group allocations until the time of assignment. Additionally, blinding was used during outcome assessments, where the assessors were blinded to group assignments to reduce any potential bias in evaluating the results. The intervention group received SST through a single one-hour session followed by weekly sessions for a month, while the control group received conventional teaching.

A power analysis was conducted prior to the study to determine the appropriate sample size. The total sample size of 121 participants was calculated to provide sufficient statistical power (80%) to detect a meaningful difference between the two groups, with an alpha level of 0.05. This calculation was based on the expected effect size of SST on clinical thinking skills, as measured by the Mini-CEX and EPAs.

Regarding statistical analysis, normality of the data was tested using the Shapiro-Wilk test. For normally distributed data, t-tests were used for comparisons between groups, while non-normally distributed data were analyzed using non-parametric tests. Chi-square tests were employed to examine categorical variables. This approach ensures that the assumptions for the chosen statistical tests were appropriately met.

### Inclusion criteria

(1) Residents with 2 years of clinical rotation experience; (2) Attendance rate of ≥ 90% during the emergency medicine rotation; (3) No major psychological issues, such as anxiety or depression; (4) Complete clinical thinking assessment results; (5)Voluntary participation with signed informed consent.

#### Exclusion criteria

1. Residents who had suspended their emergency medicine rotation due to personal leave, sick leave, maternity leave, etc.
2. Residents who did not participate in the clinical thinking assessment.
3. Incomplete teaching evaluation results.
4. Residents who had prior knowledge of the content of the simulation teaching cases.
5. Pregnant or breastfeeding residents; (6) Withdrawal from the study for other reasons.

### Teaching methods

#### Instructor Preparation

All instructors were senior faculty members involved in standardized residency training, holding at least a Master’s degree and having a mid-level or higher professional title. They had also undergone systematic training in SST and were capable of independently guiding residents.

In the SST, the roles of family members and nurses were played by teaching assistants who were familiar with the teaching objectives. The patients were high-fidelity mannequins (provided by Jucheng Teaching Technology Development Co., Ltd.), with vital signs controlled and monitored by the instructors using an external monitoring system.

#### Pre-class preparation

Instructors distributed learning materials related to critical illness assessment and the clinical management of hypovolemic shock via online resources (videos, handouts, slides, etc.) and set up pre-class quizzes. Residents were required to independently study clinical knowledge and complete a clinical thinking assessment. Critical illness assessments were based on the Airway-Breathing-Circulation (ABC) principle commonly used in clinical practice, which evaluates airway integrity, respiration, and circulation status [5]. The clinical management of hypovolemic shock followed the relevant guidelines [6].Residents were grouped into teams of three using a digital random number table, and a team leader was selected to make overall decisions, assign tasks, and lead the team.

#### Setting of teaching objectives

Both the intervention and control groups received 1 h of instruction. The goal was to apply the ABC principle to identify early signs of shock and correctly carry out the emergency management process for shock.

### Control group teaching process

#### In-class interactive discussion

Residents analyzed and interacted around the theoretical knowledge and case studies learned before class, sharing their understanding of the case and proposed management plans. They discussed their decision-making processes, fostering teamwork and communication skills. The instructor introduced the teaching objectives and guided the discussion in class, posing key questions to stimulate residents’ thinking and correct mistakes, ensuring that residents deeply understood the theoretical knowledge and were able to integrate it with practice.

#### Post-class summary and evaluation

Residents wrote a summary report based on the class discussion, highlighting the core points of the group discussion and further reflecting on their decision-making processes. The instructor graded the residents’ reports, providing targeted feedback and offering additional learning resources or problem analysis to help residents further consolidate their knowledge and improve their clinical thinking abilities.

### Intervention group teaching process

#### In-class scenario simulation and debriefing

The instructor first introduced the teaching objectives and the environment for the simulation-based course. Then, a 25-minute scenario simulation was conducted, with the participation of the patient’s family members, nurses, and residents. The family members and nurses were responsible for advancing the storyline, while the residents collaborated to adjust treatment plans in response to changes in the patient’s condition, as shown in Table 1. After the scenario simulation, the instructor and teaching assistants conducted a debriefing, addressing the issues encountered by the residents during the simulation and providing explanations and feedback.

**Table 1:** Situational simulation plot and key evaluation points

	Scene 1	Scene 2	Key Evaluation Points
High-Fidelity Mannequin	Vital signs monitoring: Blood pressure 95/56 mmHg, heart rate 109 bpm, respiratory rate 18 bpm, oxygen saturation 96%	The patient suddenly vomits 200mL of blood, experiences coughing, shortness of breath, and SpO2 drops to 83%; blood pressure drops to 75/45 mmHg, heart rate increases to 130 bpm; consciousness gradually fades, unresponsive to calling.	Critical illness assessment of residents
Patient’s Family	“Why does my father need to be intubated? What’s wrong with him?”	Family member breaks down crying: “He was fine just now!” “Doctor, what exactly is wrong with my father?”	Doctor-patient communication skills of residents
Nursing Staff	“Does the patient need to be intubated?” “What medical orders need to be given?”	① “Oxygen saturation is decreasing” ② “Is the intubation process properly prepared?” ③ “What measures need to be taken for the continued drop in blood pressure?”	Clinical treatment ability of residents with hypovolemic shock

#### Post-class evaluation and personalized feedback

Residents summarized within their groups, discussing the issues encountered during the simulation, reviewing the decision-making process and takeaways, and proposing improvement plans. After the course, the instructor evaluated the residents’ learning outcomes through a test and provided detailed feedback on their summaries and reflective reports, helping the residents identify areas for improvement and refine their skills.

### Observational indicators

#### Clinical thinking assessment scores

Residents underwent clinical thinking assessments before and after the teaching courses, with their scores serving as objective evaluation metrics for different teaching models. The assessment included seven aspects: history-taking summary (15 points), diagnosis and rationale (15 points), differential diagnosis key points (15 points), treatment principles and measures (15 points), professional knowledge questions (15 points), ethics, humanities, and professional behavior (15 points), and communication and expression abilities (10 points), for a total of 100 points. This scoring sheet is specifically used for the standardized residency training graduation assessment and has demonstrated good validity and reliability [7].

#### Entrustable Professional Activities (EPAs) scores

The EPAs evaluation was conducted via a questionnaire survey, with all instructors receiving standardized training beforehand to ensure uniformity in the evaluation criteria. The reliability of the scale in other studies was reported to be 0.8 or higher [8]. Two clinical instructors independently assessed the same resident within 2 weeks after the course, and the average score was taken as the final EPA score. The evaluation covered 9 dimensions of clinical practice, including history-taking, physical examination, diagnosis and differential diagnosis, treatment decisions, critical illness recognition and management, doctor-patient communication, teamwork, humanistic care, and self-directed learning ability [9]. Each dimension was rated on a 1–5 scale, with a total score of 45 points, as shown in Table 2. Additionally, the EPA dimension compliance rate was calculated, which refers to the percentage of EPAs with scores ≥ 4.

**Table 2:** Entrustment level distribution

Entrustment Level	Description
1	Indicates that the resident is unable to complete the specific professional activity under the direct supervision of a senior physician.
2	Indicates that the resident can complete the specific professional activity under the full, direct, and active supervision of a senior physician.
3	Indicates that the resident can complete the specific professional activity under the passive supervision of a senior physician.
4	Indicates that the resident can independently complete the specific professional activity.
5	Indicates that the resident can supervise and guide others in completing the specific professional activity.

#### Mini-CEX scores

Two weeks after the course, residents were evaluated using the Mini-CEX in real clinical settings by their instructors, who had received systematic Mini-CEX evaluation training [9]. During the assessment, the resident had 20 min to perform diagnostic and therapeutic actions, after which the instructor provided a 10-minute live feedback session. The Mini-CEX scoring sheet used a 5-point scale: very unsatisfactory (1 point), unsatisfactory (2 points), satisfactory (3 points), good (4 points), and excellent (5 points). The assessment covered 17 items across 6 dimensions: patient assessment and history-taking, physical examination, diagnosis and clinical thinking, clinical decision-making and management, communication skills and patient management, and patient monitoring and follow-up management, for a total of 85 points, as shown in Table 3.

**Table 3:** Mini-CEX evaluation criteria

Evaluation Item	Scoring Criteria
1. Patient Assessment and History Taking	A. Complete history taking, inquire about symptoms and history of gastrointestinal bleeding, including key information (such as hematemesis, melena, and past medical history)
	B. Assess the amount of bleeding and severity, and determine if emergency intervention is required
	C. Key questions: inquire about the onset, duration, triggers, and other characteristics of the symptoms
2. Physical Examination	A. Complete physical examination
3. Diagnosis and Clinical Thinking	A. Reasonable diagnostic thinking, forming a clear diagnostic approach
	B. Able to make a preliminary diagnosis based on physical examination and clinical manifestations, and distinguish between different types of gastrointestinal bleeding
	C. Select appropriate diagnostic methods to confirm the diagnosis and consider the patient’s comorbidities
	D. Timely arrange necessary examinations (e.g., endoscopy, blood tests) and ensure the rational sequence of tests
4. Clinical Decision-Making and Management	A. Timely implementation of emergency measures to ensure basic life support for the patient
	B. Correct use of medications, assessing indications and dosages
	C. Determine the need for emergency endoscopic treatment or surgery, and decide if referral or surgical options are appropriate
5. Communication Skills and Patient Management	A. Communicate the patient’s condition clearly and concisely with the patient and family, provide treatment plans, and explain related risks
	B. Efficient communication with team members to ensure smooth execution of the treatment plan
	C. Provide appropriate emotional support to reassure the patient or family members
6. Patient Monitoring and Follow-Up Management	A. Timely monitoring of the patient’s vital signs, laboratory results, and making necessary adjustments
	B. Arrange follow-up visits and plans to ensure proper management and examination after discharge
	C. Document the course of the illness, treatment plan, and treatment outcomes

#### Resident satisfaction survey

A questionnaire survey was conducted to collect feedback from residents, including their satisfaction with 5 aspects: course content, course resources, classroom interaction, teaching methods, and learning burden. A 5-point scale was used for scoring: very dissatisfied (1 point), dissatisfied (2 points), neutral (3 points), satisfied (4 points), and very satisfied (5 points), with a total score of 25 points [10].

#### Statistical methods

The reliability of the scale was calculated using SPSS 23.0 (SPSS, Inc., Chicago, IL, USA). Measurement data that conformed to a normal distribution were expressed as mean ± standard deviation, while count data were described using frequency (%). A paired t-test was employed for pre-post within-group comparisons to assess the changes over time within each group. For between-group comparisons, an independent t-test was used for continuous variables, and a chi-square test was applied for categorical data. In addition to statistical significance, Cohen’s d was calculated to estimate the effect size for the main continuous outcomes (e.g., total clinical thinking score), providing a more informative measure of the intervention’s impact. A *P* value < 0.05 was considered statistically significant.

## Results

### Comparison of general clinical data

There were no statistically significant differences between the two groups in general clinical data (*P* > 0.05), indicating comparability, as shown in Table 4.

**Table 4:** Comparison of general data

Group	Gender (Male/Female)	Age	Undergraduate	Postgraduate	Emergency Specialty	Non-Emergency Specialty
Intervention Group (*n* = 57)	33/24	25.33 ± 3.29	36 (63.16%)	21 (36.84%)	14 (24.56%)	43 (75.44%)
Control Group (*n* = 64)	40/24	24.95 ± 3.39	48 (75.00%)	16 (25.00%)	20 (31.25%)	44 (68.75%)
*t/χ2* value	0.605	1.558	-	0.158	0.414	-
*P* value	0.710	0.122	-	0.112	0.427	-

### Clinical thinking assessment scores

Intra-group comparisons showed that both groups had significantly higher clinical thinking scores after the course compared to before. However, in the dimension of ethics, humanities, and professional behavior, there was no significant difference between pre- and post-assessment in the control group (*P* > 0.05).

Inter-group comparisons revealed no statistically significant differences in clinical thinking scores or scores in any dimension between the intervention and control groups before the course (*P* > 0.05). After the course, the intervention group showed significantly higher total clinical thinking scores and scores in all dimensions compared to the control group, with statistically significant differences (*P* < 0.05), as shown in Table 5. Furthermore, in the intervention group, residents at different stratification levels had significantly higher post-course clinical thinking scores compared to the control group, with statistically significant differences (*P* < 0.001), as shown in Table 6.

**Table 5:** Comparison of clinical thinking Scores

	Total Score		History-Taking Summary		Diagnosis and Rationale		Differential Diagnosis Key Points	
	Pre-course	Post-course	Pre-course	Post-course	Pre-course	Post-course	Pre-course	Post-course
Intervention Group (*n* = 57)	68.58 ± 3.24	91.89 ± 3.00*	11.74 ± 0.70	13.37 ± 1.22*	11.49 ± 0.89	13.63 ± 1.28*	8.82 ± 1.34	13.75 ± 1.21*
Control Group (*n* = 64)	68.32 ± 3.18	80.30 ± 4.07*	11.48 ± 0.82	12.61 ± 2.19*	11.33 ± 0.76	12.28 ± 2.02*	9.05 ± 1.55	12.52 ± 1.32*
*t* value	0.429	17.675	1.820	2.313	1.090	4.322	0.840	5.347
*P* value	0.669	<0.05	0.071	0.022	0.278	<0.05	0.402	<0.05

	Treatment Principles and Measures		Professional Knowledge		Ethics, Humanities, and Professional Behavior		Summary and Communication Skills	
	Pre-course	Post-course	Pre-course	Post-course	Pre-course	Post-course	Pre-course	Post-course
Intervention Group (*n* = 57)	8.82 ± 1.50	13.75 ± 1.21*	8.79 ± 1.76	13.75 ± 1.21*	11.47 ± 0.73	14.09 ± 0.93*	7.44 ± 0.91	9.39 ± 0.73*
Control Group (*n* = 64)	9.02 ± 1.64	12.52 ± 1.32*	8.89 ± 1.75	12.52 ± 1.32*	11.25 ± 0.59	11.31 ± 1.28	7.31 ± 0.75	9.13 ± 0.93*
*t* value	0.666	8.353	0.316	15.529	1.854	13.469	0.835	1.700
*P* value	0.507	<0.05	0.753	<0.05	0.066	<0.05	0.405	<0.05

*Compared with the Pre-course statistically significant difference, *P*<0.05

**Table 6:** Post-course clinical thinking assessment scores comparison

Undergraduate	Postgraduate	Emergency	Non-Emergency
Intervention Group (*n* = 57)	90.02 ± 2.96 (*n* = 36)	91.71 ± 3.13 (*n* = 21)	92.00 ± 2.96 (*n* = 14)
Control Group (*n* = 64)	80.23 ± 3.94 (*n* = 48)	80.50 ± 4.54 (*n* = 16)	80.20 ± 4.32 (*n* = 20)
*t* value	15.016	8.885	8.843
*P* value	<0.001	<0.001	<0.001

### Comparison of EPAs scores and compliance rates

The intervention group showed significantly higher EPAs scores and compliance rates in the dimensions of history-taking, physical examination, diagnosis and differential diagnosis, treatment decisions, recognition and management of critical illness, doctor-patient communication, teamwork, humanistic care, and self-directed learning ability compared to the control group, with statistically significant differences (*P* < 0.001) (Table 7).

**Table 7:** Comparison of EPAs scores ($\:\stackrel{-}{\text{x}}$±±s)

Item	Intervention Group (*n* = 57)	Control Group (*n* = 64)	t value	*P* value
History-Taking Ability	4.18 ± 0.80	3.48 ± 1.01	4.135	<0.001
Physical Examination Ability	4.33 ± 0.61	3.63 ± 1.03	4.531	<0.001
Diagnosis and Differential Diagnosis Ability	4.26 ± 0.67	3.56 ± 0.94	4.669	<0.001
Treatment Decision-Making Ability	4.49 ± 0.63	3.50 ± 0.76	7.779	<0.001
Critical Illness Recognition and Management Ability	4.40 ± 0.53	4.04 ± 0.93	2.543	<0.001
Doctor-Patient Communication Ability	4.51 ± 0.50	3.98 ± 0.78	4.305	<0.001
Teamwork Ability	4.39 ± 0.59	3.59 ± 0.85	5.888	<0.001
Humanistic Care Ability	4.28 ± 0.62	3.58 ± 0.79	5.383	<0.001
Self-Directed Learning Ability	4.51 ± 0.57	4.23 ± 0.90	1.968	<0.001
EPAs Compliance Rate (n, %)				
History-Taking Ability	51(89.47)	27(42.19)	29.427	<0.001
Physical Examination Ability	53(92.98)	30(46.88)	29.752	<0.001
Diagnosis and Differential Diagnosis Ability	52(91.23)	26(40.63)	33.700	<0.001
Treatment Decision-Making Ability	53(92.98)	32(50.00)	26.650	<0.001
Critical Illness Recognition and Management Ability	56(98.25)	48(75.00)	13.491	<0.001
Doctor-Patient Communication Ability	57(100.00)	50(78.13)	14.100	<0.001
Teamwork Ability	54(94.74)	27(42.19)	37.621	<0.001
Humanistic Care Ability	52(91.23)	29(45.31)	28.722	<0.001
Self-Directed Learning Ability	55(96.49)	54(84.38)	4.954	0.026

### Comparison of Mini-CEX scores

The intervention group had a significantly higher total Mini-CEX score compared to the control group. In all the dimensions of the Mini-CEX, except for 4-C and 6-A, the intervention group scored significantly higher than the control group, with statistically significant differences (*P* < 0.01), as shown in Table 8.

**Table 8:** Comparison of Mini-CEX scores

Item	Intervention Group (*n* = 57)	Control Group (*n* = 64)	t value	*P* value
Patient Assessment and History-Taking				
1-A	3.96 ± 0.91	3.28 ± 1.12	3.665	< 0.001
1-B	3.96 ± 0.78	3.17 ± 1.15	4.391	< 0.001
1-C	3.86 ± 0.88	3.13 ± 1.21	3.775	< 0.001
Physical Examination				
2-A	4.07 ± 0.68	2.78 ± 0.95	8.492	< 0.001
Diagnosis and Clinical Thinking				
3-A	4.07 ± 0.80	3.58 ± 1.02	2.928	< 0.001
3-B	4.18 ± 0.71	3.47 ± 0.96	4.560	< 0.001
3-C	3.98 ± 0.74	3.05 ± 1.19	5.119	< 0.001
3-D	3.82 ± 0.78	2.95 ± 1.12	4.908	< 0.001
Clinical Decision-Making and Management				
4-A	4.05 ± 0.77	3.58 ± 1.00	2.894	0.005
4-B	3.82 ± 0.73	2.56 ± 0.89	8.452	< 0.001
4-C	3.60 ± 0.88	3.27 ± 1.12	1.793	0.076
Communication Skills and Patient Management				
5-A	3.77 ± 0.98	3.17 ± 1.16	3.047	0.003
5-B	3.96 ± 0.89	3.13 ± 1.25	4.208	< 0.001
5-C	3.67 ± 0.89	2.86 ± 1.05	4.521	< 0.001
Patient Monitoring and Follow-Up Management				
6-A	3.19 ± 1.09	3.03 ± 1.35	0.716	0.475
6-B	3.51 ± 1.09	2.88 ± 1.11	3.172	0.002
6-C	3.32 ± 1.07	2.78 ± 1.12	2.675	0.009
Mini-CEX Total Score	64.81 ± 10.57	52.66 ± 12.89	5.627	< 0.001

### Resident satisfaction survey results

Residents in the intervention group reported significantly higher satisfaction with the course content, course resources, classroom interaction, and teaching methods compared to the control group (*P* < 0.05). There was no statistically significant difference in the learning burden between the two groups (*P* > 0.05), as shown in Table 9.

**Table 9:** Comparison of resident satisfaction survey results

Item	Intervention Group (*n* = 57)	Control Group (*n* = 64)	t value	*P* value
Course Content	4.37 ± 0.49	4.00 ± 0.78	3.082	0.003
Course Resources	4.53 ± 0.50	3.78 ± 0.74	6.366	< 0.001
Classroom Interaction	4.63 ± 0.49	4.00 ± 0.80	5.183	< 0.001
Teaching Methods	4.81 ± 0.40	4.25 ± 0.73	5.095	< 0.001
Learning Burden	4.16 ± 0.77	4.33 ± 0.64	1.320	0.189

## Discussion

With the deepening of medical education reforms, traditional teaching models in emergency medicine residency training have gradually shown their limitations, especially in cultivating the rapid judgment and decision-making abilities required for the management of critically ill and emergency patients [11]. As an innovative educational approach, SST provides immersive training for residents by replicating highly realistic clinical environments. On one hand, it helps residents systematically master standardized admission processes; on the other hand, it uses an immediate feedback mechanism to expose knowledge gaps in clinical practice [12]. This teaching method not only significantly improves residents’ situational awareness and problem-solving efficiency but also reduces the operational error rate in real clinical practice through repeated exercises [13]. Given the high-risk nature and clinical complexity specific to critical illness and emergency patients, this study focuses on exploring the impact of SST on shaping residents’ clinical thinking abilities.Similar findings have been reported in previous studies using high-fidelity simulation in nursing and medical education. For example, Toqan [14] found that simulation significantly improved students’ performance, satisfaction, and self-confidence. Similarly, Jawabreh [15] demonstrated that high-fidelity simulation enhanced empathy, self-awareness, and clinical practice skills. These results align with the present study, confirming that simulation-based teaching effectively strengthens learners’ confidence, competence, and clinical reasoning.

The data from this study indicate that both groups of residents showed improvements in clinical thinking scores after training, but the intervention group exhibited significantly greater progress compared to the control group. This difference underscores the unique advantages of SST in medical education. By replicating a highly realistic clinical environment, simulation allows residents to engage in clinical decision-making training under zero-risk conditions and provides immediate, multi-dimensional feedback [16]. For example, in the case of gastrointestinal bleeding with hypoxemia used in this study, this method not only required residents to quickly assess the patient’s condition—such as determining indications for airway management—but also to coordinate critical procedures, such as endoscopic hemostasis, under time pressure, all while managing team resources. Unlike traditional passive lectures, SST fosters a “scenario experience—practical operation—reflection and optimization” closed-loop learning model. Supporting this, French scholars have used the Delphi method to confirm that this model is particularly well-suited to emergency medicine education. In their study, 24 technical and 20 non-technical skills, were deemed better suited for teaching through simulation [17]. Importantly, these training priorities encompass not only technical skills like endotracheal intubation but also emphasize the development of essential soft skills, such as clinical decision-making and crisis resource management.

Furthermore, the results of this study showed significant improvements in the ethics, humanities, and professional behavior dimension for the intervention group, while no significant changes were observed in the control group. This contrast highlights the impact of different teaching methods on residents’ development. Traditional teaching tends to focus on the transmission of theoretical knowledge, which can address ethical and professional behavior education; however, it often lacks practical opportunities for residents to deeply understand and navigate the complexities of ethical decision-making. In contrast, SST offers an interactive, contextual learning experience, enabling residents to engage directly in decision-making processes when confronted with real ethical dilemmas. This approach helps enhance their ethical literacy, particularly in areas such as doctor-patient communication, patient privacy protection, and medical decision-making [18].

The follow-up data collected 2 weeks after the course showed that the intervention group performed significantly better than the control group in both EPAs scores and compliance rates, with notable improvements in the total Mini-CEX score and scores across various dimensions. This indicates a significant enhancement in the clinical skills and decision-making abilities of the intervention group residents, demonstrating strong clinical competence. To better assess the overall abilities of residents in clinical practice, we incorporated EPAs and Mini-CEX for real-time evaluation. EPAs provide a comprehensive longitudinal assessment of residents’ competence in clinical skills, teamwork, and other areas [19], while Mini-CEX captures real-time performance of residents in specific clinical situations from a cross-sectional perspective [20]. The combined use of these 2 evaluation tools allows for a holistic measurement of residents’ overall clinical competence, while also accurately capturing their real-time performance during clinical tasks, offering a scientific and multidimensional framework for evaluating clinical teaching effectiveness.

The data from this study indicate that the intervention group residents had significantly higher satisfaction scores for the course compared to the control group. This difference was particularly noticeable in the dimensions of course content, course resources, classroom interaction, and teaching methods. SST with its unique interactive and practical characteristics, creates realistic clinical scenarios, offering residents ample opportunities for hands-on practice and fostering interaction between instructors and students. This approach significantly enhanced residents’ learning motivation and classroom engagement. Moreover, there was no statistically significant difference in the learning burden between the two groups, suggesting that while SST optimized teaching effectiveness, it did not impose additional time pressure or cognitive load. This teaching model, through carefully designed course resources and real-time feedback mechanisms, ensured high-quality instruction while maintaining a reasonable learning intensity, thereby providing an ideal environment for developing residents’ clinical thinking abilities.

This study has several key limitations. First, the small sample size and single-center design limit the generalizability of the findings. Future research with a larger, more diverse sample across multiple centers is needed to validate the effectiveness of SST.Second, the two-week follow-up period restricts our ability to assess the long-term retention of clinical skills. Longer follow-up studies are needed to evaluate how well SST’s benefits are maintained over time and applied in clinical practice.Third, the study used only a single clinical scenario (hypovolemic shock), which may limit the findings’ scenario-specific applicability. Future studies should examine multiple scenarios to assess SST’s broader effectiveness.Finally, the study did not account for individual differences among residents (e.g., prior experience or learning styles), which may influence SST’s impact. Additionally, the reliance on instructors’ subjective evaluations could introduce bias, suggesting the need for more objective assessment methods or multiple raters in future studies.

In conclusion, the results of this study demonstrate that SST significantly improves residents’ clinical thinking abilities and clinical competence. By providing opportunities to simulate real clinical scenarios, it helps residents better handle complex medical situations and enhances their emergency response skills in actual practice.

## Data Availability

Data is provided within the manuscript.
